# Addressing Water Contamination and Associated Health Issues through Community-Based Interventions: A Case Study in Khon Kaen Province

**DOI:** 10.3390/ijerph21060729

**Published:** 2024-06-04

**Authors:** Weerakanya Maneeprakorn, Gamolwan Tumcharern, Suwussa Bamrungsap, Kantapat Chansaenpak, Khoonsake Segkhoonthod, Chanoknan Rattanabut, Kullavadee Karn-orachai, Aroonsri Ngamaroonchote, Prapaporn Sangkaew, Pornpimol Wongsuwan, Dechnarong Pimalai, Nararat Yong, Tik Ouiram, Peraya Phattrapornpisit, Aurachat Lert-itthiporn, Satita Gerdsapaya, Nuttaporn Pimpha, Eknarin Thanayupong, Pitak Ngammuangtueng, Sopita Rattanopas, Pornthip Piyanuch, Preeyanut Butmee, Phongthep Noipitak, Thitiya Bunsri, Armote Somboonkaew, Sirajit Rayanasukha, Uayphorn Wannason, Sataporn Chanhorm, Kosom Chaitavon, Mongkol Thananawanukul, Ubon Cha’on, Sirirat Anutrakulchai, Deanpen Japrung

**Affiliations:** 1 National Nanotechnology Center (NANOTEC), National Science and Technology Development Agency (NSTDA), Thailand Science Park, Pathumthani 12120, Thailand; gamolwan@nanotec.or.th (G.T.); suwussa@nanotec.or.th (S.B.); kantapat.cha@nanotec.or.th (K.C.); khoonsake@ayinnova.com (K.S.); chanoknan.rat@ncr.nstda.or.th (C.R.); kullavadee.kar@nanotec.or.th (K.K.-o.); aroonsri@nanotec.or.th (A.N.); nararat.yon@nanotec.or.th (N.Y.); aurachat.ler@ncr.nstda.or.th (A.L.-i.); satita.ger@ncr.nstda.or.th (S.G.); nuttaporn@nanotec.or.th (N.P.); sopita.rat@ncr.nstda.or.th (S.R.); pornthippiyanuch@gmail.com (P.P.); preeyanutbutmee@gmail.com (P.B.); phongthep.noi@ncr.nstda.or.th (P.N.); thitiya.bsri@gmail.com (T.B.); 2 National Electronics and Computer Technology Center, National Science and Technology Development Agency (NSTDA), Thailand Science Park, Pathumthani 12120, Thailand; armote.somboonkaew@nectec.or.th (A.S.); sirajit.ray@nectec.or.th (S.R.); uayphorn.wan@nectec.or.th (U.W.); sataporn.chanhorm@nectec.or.th (S.C.); kosom.chaitavon@nectec.or.th (K.C.); 3 10th Environment and Pollution Control Office (Khon Kaen), Ministry of Natural Resources and Environment, Khon Kaen 40000, Thailand; mkenv_kk@yahoo.com; 4 Chronic Kidney Disease Prevention in the Northeast of Thailand (CKDNET), Khon Kaen University, Khon Kaen 40002, Thailand; ubocha@kku.ac.th; 5 Department of Biochemistry, Faculty of Medicine, Khon Kaen University, Khon Kaen 40002, Thailand; 6 Department of Internal Medicine, Faculty of Medicine, Khon Kaen University, Khon Kaen 40002, Thailand

**Keywords:** learning innovation platform, water contamination, community-based interventions, public health, heavy metals, chronic kidney disease

## Abstract

A recent study conducted in Khon Kaen Province, Thailand, evaluated the effectiveness of a technology-assisted intervention aimed at improving water quality and addressing related health issues in communities around key water bodies. The intervention targeted health concerns associated with water contamination, including chronic kidney diseases, skin conditions, hypertension, and neurological symptoms. The study included water quality assessments and health evaluations of 586 residents and implemented a Learning Innovation Platform (LIP) across 13 communities. Results showed significant improvements in the community, including a decrease in hypertension and skin-related health issues, as well as enhanced community awareness and proficiency in implementing simple water quality assessments and treatment. The study demonstrated the value of a comprehensive, technology-driven community approach, effectively enhancing water quality and health outcomes, and promoting greater community awareness and self-sufficiency in managing environmental health risks.

## 1. Introduction

**Figure 1:**
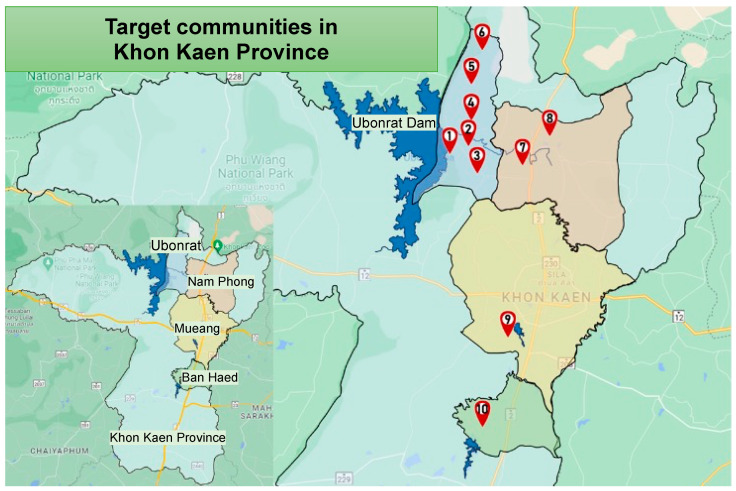
A geographical representation of ten targeted sub-districts in Khon Kaen province. The sub-districts are numerically labeled from 1 to 10 and are categorized based on their proximity to specific water bodies. Eight sub-districts, indicated as (1) Khaeun Ubonrat sub-district, (2) Thung Pong sub-district, (3) Khok Sung sub-district, (4) Ban Dong sub-district, (5) Na Kham sub-district, (6) Si Suk Samran sub-district, (7) Kud Nam Sai sub-district, and (8) Nam Phong sub-district, are situated around the Ubonrat Dam and Lam Nam Phong River. Additionally, one area around Kaeng Nam Ton Pond is represented by (9) Don Chang sub-district, and one area around Kaeng La Wa Pond is represented by (10) Khok Samran sub-district.

Water contamination stands as a critical public health issue, contributing significantly to a range of health disorders such as chronic kidney disease (CKD), skin ailments, hypertension, gastrointestinal complications, and neurologic symptoms [1,2]. The presence of hazardous chemical pollutants in water sources amplifies the risk of long-term health effects [3]. This crisis is particularly acute in northeastern Thailand, where this study targets ten specific sub-districts within Khon Kaen province (Figure 1) to investigate these concerns.

Critical water reservoirs, such as Ubonrat Dam and Lam Nam Phong River, are crucial for sustaining the socioeconomic structure of Northern Khon Kaen. Ubonrat Dam plays a vital role in providing electricity and supporting agriculture, and aquaculture. It intercepts the Lam Nam Pong River, which serves as a lifeline for the Nam Phong District’s 12 sub-districts and 168 villages/communities [4]. These reservoirs are not only essential for water provision but are also major tourist sites in the region. Similarly, Kaeng La Wa and Kaeng Nam Ton Pond support local agriculture and household water, enriched by their connection to the Chi River.

Recent studies have demonstrated the efficacy of advanced filtration systems in significantly reducing contaminants such as heavy metals and pesticides in drinking water. Multi-stage filtration processes, including activated carbon filters, reverse osmosis, and UV sterilization, have shown substantial improvements in water quality and public health outcomes. Additionally, community-based monitoring programs have been successful in various regions. These programs train local residents to regularly test and monitor water quality, fostering a sense of ownership and responsibility toward maintaining safe water sources. Public health campaigns have also played a crucial role in educating the public about the dangers of contaminated water and promoting safe water consumption practices. These initiatives have helped reduce the incidence of waterborne diseases and improve overall community health [3,4].

Despite these advances, several critical questions remain unanswered. Most existing studies have focused on short-term interventions without addressing long-term sustainability and community self-sufficiency. Additionally, there is a lack of comprehensive studies that simultaneously address multiple contaminants and their combined health effects. Many interventions have also failed to integrate local knowledge and practices, essential for the successful implementation and acceptance of water quality improvement measures.

Initiated in 2019, our research aims to shed light on water consumption and potential contaminants affecting health around these critical water bodies. We surveyed 1036 residents and performed in-depth analyses of water and sediment samples. The tests included examinations for metals, heavy metals, pesticides, and herbicides, and identifying contaminants that could be causally linked to local health issues [4]. Consequently, we focused on ten sub-districts known for water quality concerns, with eight being part of our initial survey and two newly identified based on previous health research (Figure 1).

After selecting these areas, health assessments were conducted before and after implementing our interventions, focusing on symptoms and conditions indicative of water quality-related health concerns [5]. Our approach mobilized the community, training over 40 individuals through the Learning Innovation Platform (LIP) to act as local ‘innovators’ in water quality monitoring and simple purification methods [6].

**Figure 2:**
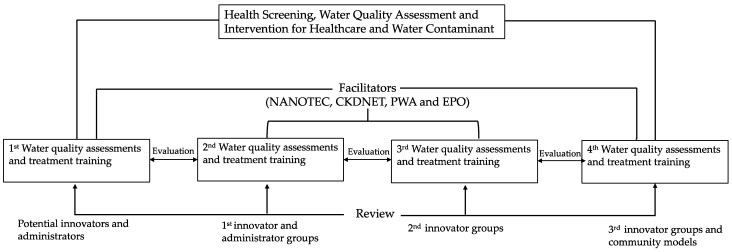
Illustration of the LIP-driven process for continuous health screening and water quality improvement, facilitated by four key organizations: NANOTEC (National Nanotechnology Center), CKDNET (Chronic Kidney Disease Prevention in the Northeast of Thailand), PWA (Provincial Waterworks Authority), and EPO (Environmental and Pollution Control Office). Each activity incorporates comprehensive assessments and training, anchored by central reviews to ensure iterative improvement and thorough oversight.

The “Learning and Innovation Platform” (LIP) is a learning program designed to foster technology and innovation implementation. This platform aims to train innovators who can effectively use our technology, knowledge, and innovation to screen and monitor hazardous chemical contaminations in water sources and community drinking water, while also facilitating the improvement of water quality. This platform involves the local community as the key actor, research teams as the facilitator, and government agencies, state enterprises, and the private sector in the community area as the network management. (Figure 2) Each participant has important roles and responsibilities in designing and implementing learning activities at various levels to support skill and knowledge development in society and organizations comprehensively and systematically. In measuring the effectiveness of activities, the innovation learning levels of innovators were assessed as follows: Level 1—able to expand and link community resources continuously, Level 2—capable of researching and generating knowledge, technology, and innovation, Level 3—able to disseminate knowledge, technology, and innovation, and Level 4—capable of receiving, adapting, and applying technology and innovation.

The core objective of this research is to study the effectiveness of these interventions. We intend to compare community health metrics before and after implementing surveillance and remediation measures. Key health indicators, such as CKD rates, will provide an all-encompassing view of the interventions’ impact on water quality and public health [7]. Through this comprehensive strategy, the study aims to establish a community-based model to effectively combat water contamination. We envision this model serving as a scalable, sustainable framework capable of informing national policy [8].

## 2. Materials and Methods

### 2.1. Identification of Study Areas

Before conducting any surveys or water quality assessments, a comprehensive meeting was held to identify and finalize the study areas. The meeting brought together representatives from various key stakeholders involved in the regions surrounding the Ubonrat Dam and Lam Nam Phong River. Attendees included representatives from the Khon Kaen governor’s office, the Provincial Administrative Organization, the Sub-district Administrative Organization, and local companies and factories. Additionally, medical specialists, including nephrologists and public health physicians from the Chronic Kidney Disease Prevention in the Northeast of Thailand (CKDNET) and Khon Kaen University, also presented to provide specialized insights. Collectively, these stakeholders deliberated on the specific areas that would serve as the focus of this study, keeping in mind the objectives and the scope of the research.

### 2.2. Survey Design

#### 2.2.1. Questionnaire Survey

The questionnaire was crafted to gather insights into water consumption patterns, health conditions, and public awareness about water quality. Before administration, the survey underwent a pre-test to confirm its clarity, relevance, and suitability. Ethical approval for the study was secured under code HE601035, granted by the Office of the Khon Kaen University Ethics Committee in Human Research (Institutional Review Board number IRB00001189), located at Khon Kaen University, Thailand. A total of 1036 residents were randomly selected to participate in the survey. Informed consent was obtained from all participants before their involvement in the study.

#### 2.2.2. Water Contaminant Survey

Concurrent with the questionnaire, water and sediment samples were gathered from various locations within the designated study areas. In the case of the Lam Nam Phong River, samples comprising both water and sediment were collected at 5 km intervals along the river. This approach aimed to mitigate bias and prevent the unfair attribution of contamination to specific communities or stakeholders. The samples were rigorously tested for an array of potential contaminants, following standardized procedures to ensure the validity and reliability of the results. These tests targeted metals such as aluminum (Al), chromium (Cr), manganese (Mn), iron (Fe), cobalt (Co), copper (Cu), arsenic (As), cadmium (Cd), and lead (Pb). For pesticide analysis, substances like chlorpyrifos, glyphosate and its metabolite (aminomethyphosphonic acid), 2,4-dichlorophenoxyacetic acid (2,4-D), paraquat dichloride, and bispyribac sodium were examined. By adhering to these procedures, the study sought to provide a comprehensive assessment of water quality, focusing on contaminants with known or suspected health implications. The sampling and measurement of heavy metal contamination and pesticides in sediment and water along Lam Nam Phong River and Ubonrat Dam followed the processes and standard methods of the Pollution Control Department, Ministry of Natural Resources and Environment, Thailand, 2018. These methods reference international standards: ISO 5667-6:2014—Water quality—Sampling—Part 6: Guidance on sampling of rivers and streams [9], ISO 5667-12:2017—Water quality—Sampling—Part 12: Guidance on sampling of bottom sediments [10], and US EPA Standard Operating Procedures for Surface Water Sampling (U.S. EPA, 2023) [11].

#### 2.2.3. Data Analysis

The amassed data were subjected to rigorous statistical analysis using Excel and Origin (Version 6.0) software. This process facilitated data cleaning and enabled the identification of patterns related to water quality and health outcomes within the local community.

### 2.3. Criteria for Identifying Target Areas for Training Local Innovators and Deploying Water Quality Interventions

Study areas were selected based on a comprehensive approach that considered four key criteria: (1) existing infrastructure and prior relationships with the community; (2) preliminary assessments of community interest, leadership strength, and willingness to participate, gathered through surveys and interviews; (3) pre-existing health and water contamination data; and (4) geographical and environmental conditions, specifically the communities’ use of diverse water sources such as Ubonrat Dam, Lam Nam Phong River, Kaeng Nam Ton, and Kaeng La Wa Pond. This comprehensive set of criteria ensured a well-informed selection of study areas conducive to implementing water quality innovations. The sampling and measurement of heavy metal contamination in water samples collected from ten community-operated water treatment plants adhered to the processes and standard methods of the Thailand Provincial Waterworks Authority. These procedures align with the guidelines outlined in the World Health Organization’s “Guidelines for Drinking-Water Quality,” 4th edition (2011) [12]. All samples were analyzed by a laboratory accredited with the ISO/IEC 17025 standard [13], ensuring that the analytical results are reliable and meet international quality and technical competency criteria.

### 2.4. Pre- and Post-Intervention Health Assessments to Evaluate LIP and Water Quality Improvements

Comprehensive health assessments were carried out on 568 residents aged 18 years or older, both before and after local innovators were trained through the Learning Innovation Platform (LIP) to implement water quality improvements (Figure 2).

These assessments took place in 10 targeted sub-districts and were facilitated in partnership with local Health Promoting Hospitals and public health volunteers. Participants underwent a standardized health questionnaire [14] along with a set of clinical laboratory tests. Blood and urine samples were collected to assess kidney and liver function, specifically evaluating levels of serum creatinine and the urine albumin-to-creatinine ratio, as well as conducting general urinalysis. Liver function was evaluated through alanine aminotransferase (ALT) tests, and a complete blood count (CBC) was performed for anemia screening. All clinical tests were conducted at Srinagarind Hospital. Each participant received individual health screening results and personalized medical advice from qualified doctors. This study was conducted following the Declaration of Helsinki and approved by the Institutional Review Board of the Office of the Khon Kaen University Ethics Committee in Human Research (Institutional Review Board number IRB00001189), Khon Kaen University, Thailand.

## 3. Results

### 3.1. Survey Results on Water Consumption and Quality Concerns

**Figure 3:**
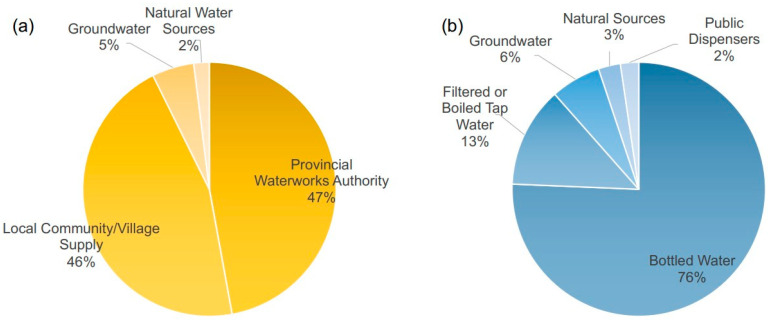
Water Sources for 1036 Residents around Ubonrat Dam and Lam Nam Pong River, 2019. (**a**) Daily water usage primarily comes from the Provincial Waterworks Authority (47.55%) and local community/village supply (45.92%), with lesser amounts from groundwater (5.42%) and natural sources (2%). (**b**) Drinking water preferences show that the majority use bottled water (75.65%), followed by filtered or boiled tap water (12.8%), groundwater (6.4%), natural sources (2.81%), and public dispensers (2.33%). Note: The percentages provided are rounded for simplicity in the chart representation.

Investigations into the water consumption habits and health perceptions of 1036 residents living near the Ubonrat Dam and Lam Nam Phong River revealed that a substantial majority of the community (92%) relied on tap water for their daily needs. This tap water was sourced either from the Provincial Waterworks Authority (47.55%) or local community/village water supply systems (45.92%). Smaller fractions of the population used groundwater (5.42%) or natural water sources (2%), as shown in Figure 3a. Despite the prevalent use of tap water, residents expressed concerns over its quality, noting attributes such as color, turbidity, foul smell, and unpleasant taste. Notably, these local water sources were tested for quality only once a year, and the results, even when they did not meet standards, were not communicated to the residents.

In addition to tap water usage, we found that most residents (75.65%) primarily rely on bottled drinking water. Only 12.8% used tap water for drinking, and even then, only after boiling or filtering it. The remaining residents used groundwater (6.4%), natural water sources like rain or well water (2.81%), and water from public dispensers (2.33%), as shown in Figure 3b. Intriguingly, much of the bottled water consumed was produced locally, using water from the Nam Phong River as the raw material. This situation underscored the need for consistent quality control and regular monitoring of both tap and bottled water sources.

### 3.2. Comprehensive Analysis of Water and Sediment Quality: Implications for Public Health and Environmental Safety

**Figure 4:**
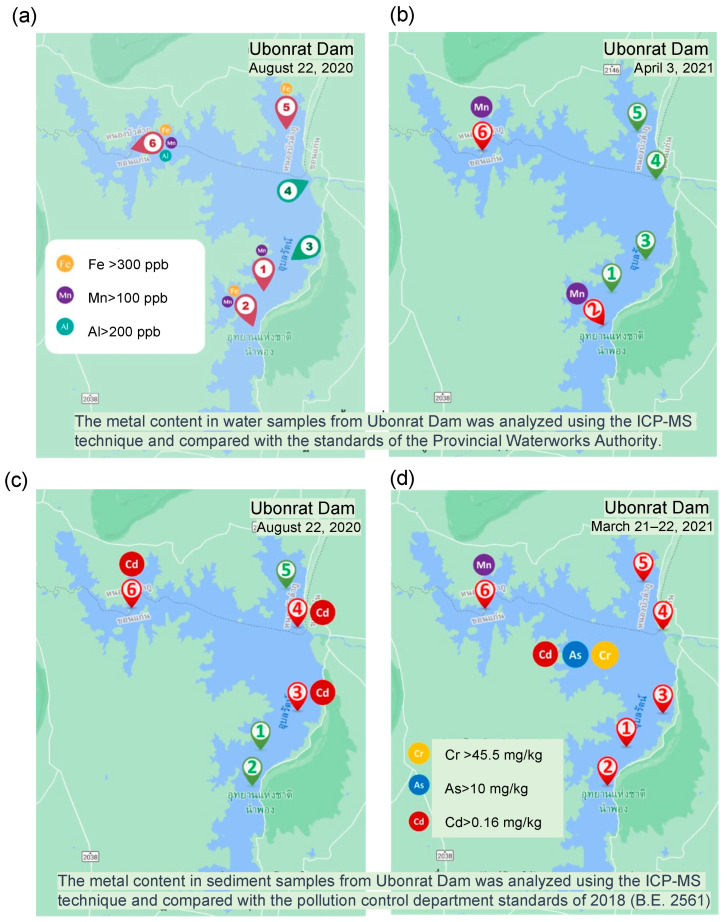
Geographic distribution of chemical contaminants around the Ubonrat Dam. The map pinpoints areas where levels of Fe (indicated in yellow), Mn (in purple), and Al (in green) in both water and sediment samples did not meet the established standards. Panel (**a**) reveals locations where water samples containing Fe, Mn, and Al failed to meet Provincial Waterworks Authority guidelines during the rainy season. Panel (**b**) similarly indicates areas during the hot season. Panel (**c**) presents locations where sediment samples containing Cr (marked in yellow), As (in blue), and Cd (in red) fell short of Pollution Control Department standards in the hot season. Panel (**d**) does the same for the rainy season. Note: The collection dates for freshwater samples in Panels (**a**,**c**) were 22 August 2020. Sediment samples for Panel (**b**) were collected on 3 April 2021, while those for Panel (**d**) were collected between 21–22 March 2021.

Upon evaluating the water and sediment quality in the surrounding area of the Ubonrat Dam and along a 35-km segment of the Lam Nam Phong River, the analysis revealed the presence of multiple chemical contaminants that exceeded the established standards for both environmental and domestic use. Specifically, Fe, Mn, and Al concentrations in water (Figure 4a,b) surpassed allowable limits for household and drinking water. Additionally, sediment samples revealed detectable levels of Cd, Cr, and As, as shown in Figure 4c,d. Seasonal variations in contaminant levels were also evident, with higher concentrations during the hot season and lower concentrations during the rainy season, as detailed in Figure 4. Notably, glyphosate was present in both water and sediment samples. Although these concentrations were within current permissible limits, their existence indicates a state of environmental contamination. It is important to note that the half-life of glyphosate can be less than 19 weeks, depending on water conditions [15], suggesting that continued use might elevate levels beyond acceptable thresholds.

**Figure 5:**
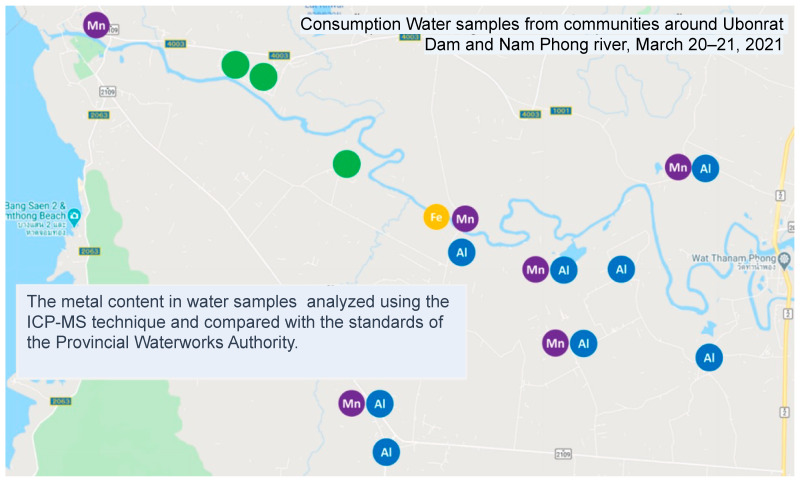
Geographic distribution of household water quality issues along the Lam Nam Pong River. This map highlights community locations where household water samples did not meet Provincial Waterworks Authority standards for Mn (indicated in purple), Al (in blue), and Fe (in yellow). Note: Water samples were collected between 20–21 March 2021.

Further analysis of household and drinking water quality around the Ubonrat Dam and Lam Nam Phong River confirmed elevated levels of Fe, Mn, and Al. Although these levels were not immediately toxic, prolonged exposure could pose potential health risks, as indicated in Figure 5.

Our water quality surveys aligned with previous health assessment surveys conducted by CKDNET on 973 residents in communities around these aquatic environments. CKDNET’s earlier studies showed that areas with chemical contaminants in both freshwater and household water sources exhibited abnormal biomarkers associated with kidney dysfunction. In three out of the eight areas examined, the incidence of chronic kidney disease (CKD) was found to be higher than 25%. Although not all areas with identified water contaminants corresponded to adverse health findings from CKDNET’s surveys, the results still raised significant concerns. These findings point to shortcomings in community-managed water treatment systems, which often operate due to the absence of Provincial Waterworks Authority service or the higher costs associated with them.

### 3.3. Seasonal Variability and Treatment Efficacy in Heavy Metal Concentrations in Community Water Treatment Plants

Water samples were collected from ten community-operated water treatment plants located in various villages around Ubonrat Dam, Lam Nam Phong River, Kaeng Nam Ton, and Kaeng La Wa Pond in Khon Kaen Province (Table 1). The samples were obtained at three distinct stages in the treatment process: from raw water before entering the plant, from the water within the precipitation container, and from the treated water ready for community distribution. These samples were analyzed for concentrations of Al, Cr, Cu, Mn, Fe, Co, Cd, As, and Pb using advanced laboratory techniques such as Atomic Absorption Spectroscopy (AAS), Inductively Coupled Plasma Mass Spectrometry (ICP-MS), and Inductively Coupled Plasma Optical Emission Spectrometry (ICP-OES). The findings are detailed in Table 2.

In this study, water samples were collected during three crucial periods: the late hot to early rainy season (16 July–8 August 2022), the mid-rainy season (29–30 August 2022), and the late rainy to early cool season (September–October 2022). Table 2 presents heavy metal concentrations at both inlet and outlet sample points for 13 community water treatment plants, with samples collected during the late hot to early rainy season. Primarily, levels of Al, Cr, Fe, Co, and Cu mostly remained within regulatory limits, except for elevated Mn concentrations at the Don Chang and Kok Nam Kliang plants. Manganese is a metal commonly found in high concentrations in surface water sources in Thailand. Although Mn concentrations appear to have decreased after treatment, they still exceed standard limits (as shown in Table 2; at the Don Chang and Kok Nam Kliang plant outlets). These indicate the efficacy of water treatment processes at the plant. Additionally, Kok Nam Kliang showed Fe levels above the permissible threshold after treatment. Notably, the increased Fe levels at this facility could not be linked to their raw water sources, as other plants with similar sources demonstrated lower levels of these metals (Table 2).

Regarding As, Cd, Pb, and Hg concentrations, the study found that all 13 water treatment plants exhibited levels of these metals that exceeded standard limits. Furthermore, minimal differences in these concentrations were observed before and after the water treatment process, suggesting that the existing treatment systems might be ineffective or inadequate in removing these metals (Table 2). Therefore, to test the hypothesis that the metal contamination might originate from the water production process, an additional water sampling point was added at the precipitation chamber of the water production system during the second and third sampling periods. These sampling periods took place during the mid-rainy season (29–30 August 2022) and the transition from the late rainy to early cool season (September–October 2022) (Table 3). During subsequent sampling in the rainy and transition-to-cool seasons, most contaminants remained within acceptable limits, except for persistent elevated Mn levels at the Kok Nam Kliang and Nong Ma Khuea plants.

Considering the findings across all three sampling phases, considerable fluctuations in Mn, As, Pb, Cd, and Hg levels were observed. While most measurements remained within regulatory guidelines during the rainy and transition-to-cool seasons (as displayed in Table 3), the study highlighted the seasonal impact on water quality. Samples collected during the transition from the late hot to the early rainy season, which marks the end of Thailand’s long dry season, showed a considerable accumulation of various contaminants in the water, resulting in high levels of detected contamination. The contamination levels gradually improved during the rainy season. These data informed the selection of appropriate technological solutions for each community, such as point-of-test sensors for Mn, As, Pb, Cd, and Cu. Additionally, the data called into question the effectiveness of current community water treatment systems in consistently reducing contaminant levels. Data from Table 3 show that the contamination might have originated from the precipitation chamber of the water production system. This is illustrated by the Mn levels detected in some sampling areas in Table 3, where no contamination was found in the inlet samples, but contamination was present in the precipitation chamber. This finding is consistent with the results for Fe and certain heavy metals in Table 2, where the contamination levels in the outlet water were higher than those in the inlet. Therefore, it is recommended that the village water supply system administrators regularly maintain the precipitation chamber.

In treatment systems that did not meet quality standards, simple filtration materials and engineering designs were integrated to enhance water quality. Additionally, it was noted from the Al concentrations listed in Table 3 that some areas exhibited high levels of Al in the precipitation chamber, while none was detected at the inlet. In other areas, higher Al levels were found at the outlet, which is the water after the quality improvement process. These Al concentrations may result from the excessive addition of alum in the coagulation process. Thus, routine turbidity and Mini Jar tests were conducted to monitor raw water clarity and optimize the use of potassium alum for precipitation. Even though they were provided with standard protocols, the community faced challenges adapting these guidelines to their specific needs. Notably, these water treatment plants are located near agricultural fields that frequently use pesticides and fertilizers. These fertilizers might contain a mix of heavy metals as inert ingredients in their formulations [16], underscoring the importance of regular monitoring and adjustments by the community.

### 3.4. Baseline Health Assessment of Community Volunteers before Water Quality Improvement and Learning Innovation Platform (LIP) Training: Demographics, Physical Measurements, and Laboratory Findings

*Characteristics of Participants:* The study included 568 volunteers with an average age of 61.6 ± 23.0 years. Of these, 156 were male (27.5%) and 412 were female (72.5%). The average weight for 527 participants (41 of whom had dropped out) was 59.9 ± 11.3 kg, and their body mass index (BMI) averaged 24.8 ± 4.25 kg/m^2^. Overweight and obesity were prevalent in 65.1% and 44% of this subgroup, respectively. The average systolic blood pressure was 138.0 ± 19.8 mmHg, and the average diastolic blood pressure was 80.7 ± 11.9 mmHg. High systolic pressure (>140 mmHg) was observed in 42.5%, while high diastolic pressure (>90 mmHg) was observed in 21% of the participants (Table 4).

*Health-Related Findings:* Of the 514 participants, the average serum creatinine level was 0.86 ± 0.29 mg/dL. The average estimated glomerular filtration rate (eGFR) was 82.3 ± 18.1 mL/min/1.73 m^2^. An abnormal eGFR (<60 mL/min/1.73 m^2^) was found in 62 participants (12.1%). Of the 511 participants tested for urine albumin-to-creatinine ratio (UACR), the average value was 7.92 mg/g (range 3.87–27.27). Elevated UACR (>30 mg/g) was found in 120 participants (23.5%). Microscopic hematuria and pyuria were present in 9.1% and 18.2% of the cases, respectively. The average alanine transaminase (ALT) level was 21.6 ± 12.2 U/L, and elevated levels suggesting hepatitis (ALT > 36 U/L) were found in 47 participants (9.14%). The average hemoglobin level was 12.7 ± 1.50 g/dL, and anemia was observed in 150 participants (29.5%).

*Prevalence of Chronic Kidney Disease:* Based on assessment through interviews, and blood and urine tests, chronic kidney disease (CKD) was estimated to be prevalent in 182 participants (32.0%). When categorized by stages, there were 59 (10.4%) in Stage 1, 61 (10.7%) in Stage 2, 54 (9.5%) in Stage 3, 7 (1.2%) in Stage 4, and 1 (0.18%) in Stage 5.

*Community Prevalence:* The prevalence of CKDu varied across different communities. Focusing on participants aged below 70, it was observed that the villages of Nong Makuea (12.5%), Don Po Daeng (14.6%), Nong Tae (15%), and Khok Nam Kliang (10%) had relatively high prevalence rates.

### 3.5. Strategy for Implementing and Monitoring Water Quality Improvement through Innovation and Technology

Following the criteria mentioned earlier for selecting target areas to train local innovators and implement water quality interventions, the Nong Tae water treatment plant was chosen as a community model. This selection was for improving water quality using LIP training combined with infrastructure enhancements in the water plant. For infrastructure improvement in the Nong Tae water plant, repairs and enhancements were focused on the existing system without making structural changes. These improvements included upgrading the raw water pump to efficiently pump water into the system, modifying the sedimentation tank for easier cleaning by using a tube settler, installing a chemical dosing system, and adding a carbon filter tank before distributing the water to users. In the LIP activities, besides providing knowledge on the maintenance of village water supply systems and water quality monitoring, we introduced equipment sets for monitoring chemicals commonly found in water. These include test kits for metals/heavy metals and pesticides, such as Mn, Cu, F, Hg, Pb, As, Glyphosate, and Paraquat. Additionally, we provided tools for assessing appropriate coagulant dosages, such as a simple turbidity measurement device and a Mini Jar Test for coagulant evaluation. We also created a manual for maintaining village water supply systems and using these tools, which was handed over to the community for their use. In contrast, other communities only underwent the LIP training program, as illustrated in Table 5.

For Ban Nong Tae, which was used as a model in the study, it was found that water quality before (Table 2) and after the infrastructure enhancements (Table 3, 3rd round) showed significant improvement. There was no contamination of heavy metals, and the amount of aluminum in the finished water (outlet), primarily resulting from improper alum dosing, was significantly reduced.

Before initiating the LIP-driven community, 527 participants from the target communities received comprehensive health checks. Physical examinations and clinical laboratory tests were conducted, and individual results were provided to participants, accompanied by medical consultations during the LIP training. A summary of these health assessments is presented in Table 4.

The initial session of the LIP played a crucial role in developing a group of trained community members. These first 10 participants went through an extensive training program, which highlighted the potential risks of water contaminants, especially those identified in local water sources and at both the inlet and outlet of community water treatment plants. This education aimed to increase awareness about the importance of using water that adheres to safety standards, thereby connecting public health to water management.

In the second LIP session, the initial cohort of 10 community innovators assumed a mentorship role, guiding the training of at least another 10 interested individuals. This transfer of knowledge and skills fostered a second generation of community innovators, as illustrated in Table 5. Throughout the LIP, continuous support, evaluations, and reviews were provided and facilitated by key local agencies. This comprehensive and iterative approach not only ensures community empowerment in water resource management but also amplifies awareness and action regarding water quality and health, making the program’s impact widespread and lasting.

After completing the LIP activities, a total of 46 innovators were developed. Among them, 17 innovators have the capability to serve as station masters or community representatives. The community innovator levels are divided into four categories based on their learning abilities: Level 1: Able to continuously expand and connect community resources (Station Master); Level 2: Able to discover and create knowledge, technology, and innovations; Level 3: Able to transfer knowledge, technology, and innovations; and Level 4: Able to receive and adapt technology and innovations.

Additionally, after the completion of the LIP training program, several positive changes were observed in the community. There was a shift in attitudes and an increase in awareness among the local population about the importance of monitoring water quality and detecting chemical contamination that could affect their health. People became more interested in tracking chemical contaminants in the water and expressed a desire to manage and maintain their community’s water supply themselves. This was evident as more village technicians and health volunteers participated in subsequent LIP activities on their own initiative.

Moreover, it was found that more residents brought water samples from their households for quality testing during the water quality monitoring stations set up alongside LIP activities. The number of water samples tested increased by 30% each time a LIP activity was held. According to surveys conducted in the model community of “Nong Tae”, residents felt that the water was cleaner and had greater confidence in the water produced by their village water supply system. As a result, they started using this water for their daily needs more frequently, reducing their reliance on bottled drinking water.

### 3.6. Evaluation of Health Indicators and Kidney Function following the Implementation of the Learning Innovation Platform (LIP)

The outcomes of the comparative health assessment before and after the introduction of the LIP-driven community are presented in Table 6. Statistically significant improvements were noted in various symptoms, including lethargy, arthralgia, muscle ache, backache, abdominal bloating, headache, loss of appetite, insomnia, cramps, jaundice, palpitations, gasping for breath, and diarrhea. Over 6 months, there was also a decrease in the prevalence of conditions such as high blood pressure, skin rashes, gastrointestinal disorders, and neurological problems. However, no statistically significant changes were observed in the overall prevalence of kidney disease or the average glomerular filtration rate after the platform’s implementation. Notably, there was a decline in urinary albumin levels, a biomarker indicating initial changes in kidney disease.

In a sub-analysis focused on kidney health and categorized by geographical areas (Table 7), significant declines in the glomerular filtration rate were notably observed in the regions of Kok Samran and Nong Makuea, Haet District, Na Kham, and Kok Namkliang. A decrease in median urinary albumin levels was detected in most regions (9 out of 13), with the exceptions being Pa Lium, Kok Samran, Nong Bua Noi, and Kam Bon. Interestingly, while considering the total number and severity of all chronic kidney disease patients, no statistically significant changes emerged post-implementation. However, significant shifts in the prevalence of idiopathic kidney diseases were observed in two specific areas: a decrease in the pilot community for water treatment in the project, Nong Tae, Dong Sub-district (from 16.22% to 2.70%), and an increase in Kam Bon, Kok Sung Sub-district (from 2.94% to 14.71%). Particularly in the area of Kam Bon, Kok Sung Sub-district, our previous survey identified Cd, Cr, and As levels that surpassed the standard limits in soil sediments.

## 4. Discussion

The LIP has proven to be an effective community-based intervention for addressing both water quality and health issues in Khon Kaen Province. The program successfully developed a training model emphasizing the implications of water contamination [17,18] and tailored its educational content to address local water management challenges [17]. Notably, the initiative led to improvements in general health markers such as sleep quality, emotional well-being, and cognitive functions [1]. Additionally, there was a significant reduction in hypertension cases [19], highlighting the combined benefits of enhancing water quality and community health education [20].

One of the primary strengths of this study is its comprehensive approach, integrating community training with rigorous health and environmental assessments. The LIP model empowered local residents to monitor water quality and implement simple purification methods, fostering community ownership and sustainability. This empowerment is crucial for ensuring long-term improvements in water quality and health outcomes.

However, the study has several limitations. The relatively short follow-up period limits the ability to assess long-term health outcomes and the sustainability of the intervention. Additionally, the study did not control for all potential confounding variables, such as dietary habits and other environmental exposures, which could influence the health outcomes observed [21]. Future research should include longer follow-up periods and more comprehensive control of confounding variables to better understand the long-term impacts of such interventions.

The elevated levels of heavy metals such as Cd, Cr, and As in soil sediments in Kam Bon and Kok Sung Sub-districts raise significant health concerns. Chronic exposure to these metals can lead to various adverse health effects [22]. Cd exposure is associated with kidney damage and an increased risk of osteoporosis. Cr, particularly hexavalent chromium, is a known carcinogen, while As exposure is linked to skin lesions, developmental effects, cardiovascular disease, neurotoxicity, and diabetes. These findings underscore the necessity for a multifaceted approach that addresses both health education and environmental remediation [23]. Strategies to mitigate these risks should include regular monitoring of environmental contaminants, community education on the sources and health effects of these metals, and interventions to reduce exposure, such as improving water treatment processes and soil remediation efforts.

## 5. Conclusions

In summary, the LIP demonstrated its efficacy as a community-based intervention tailored to address the pressing concerns of water contamination and associated health issues in Khon Kaen Province. The program has shown significant improvements in general health indicators, including reductions in hypertension and enhancements in sleep quality and emotional well-being. Additionally, there was a noted improvement in the community’s ability to monitor and manage water quality independently. However, the findings also highlight the presence of environmental contaminants such as Cd, Cr, and As, which pose significant health risks. These findings emphasize the need for ongoing environmental monitoring and remediation efforts, alongside community education and health interventions.

Future research should focus on longer-term follow-up studies to assess the sustainability of health improvements and the long-term effectiveness of the LIP model. Additionally, future studies should aim to control for a wider range of confounding variables, such as dietary habits and other environmental exposures, to provide a clearer understanding of the factors influencing health outcomes. Furthermore, research should explore the development and implementation of more advanced water treatment and soil remediation technologies to address the identified environmental contaminants effectively. By addressing these areas, future research can build on the current study’s findings and contribute to the development of more comprehensive and sustainable strategies for improving water quality and public health in communities facing similar challenges.

## Figures and Tables

**Table 1:** District, Sub-District, and village names of 15 surveyed community water treatment plants and their corresponding raw water sources.

District	Sub-District	Village/Community Name	Source of Raw Water
Mueang	Don Chang	Ban Don Chang	Kaeng Nam Ton Pond
		Ban Pa Lueam	Kaeng Nam Ton Pond
Ban Haed	Kok Samran	Kok Samran	Kaeng La Wa Pond
		Nong Makhuea	Kaeng La Wa Pond
		Don Po Daeng	Kaeng La Wa
Nam Phong	Kud Nam Sai	Nong Bua Noi	Lam Nam Phong River
	Nam Phong	Huai Suea Ten	Lam Nam Phong River
Ubonrat	Ubonrat Dam	Kok Nam Kliang	Ground water
	Sri Suk Samran	Sri Suk	Ground water
	Ban Dong	Nong Tae	Lam Nam Phong River
		Huai Sai	Lam Nam Phong River
	Tung Pong	Sub Somboon	Ground water
	Na Kham	Na Kham	Groundwater (No treatment system)
	Kok Sung	Kok Sung	Provincial Waterworks Authority
		Kham Bon	Provincial Waterworks Authority

**Table 2:** Concentrations of metals in water samples from community treatment plants collected during the transition from late hot to early rainy season (16 July–8 August 2022) (First sample collection).

Community Name	Metal Contaminant (mg/mL)																			
	Al		Cr <0.05 mg/L		Mn <0.3 mg/L		Fe <0.3 mg/L		Co		Cu <2.0 mg/L		As <0.01 mg/L		Cd <0.003 mg/L		Pb <0.01 mg/L		Hg <0.001 mg/L	
	Inlet	Outlet	Inlet	Outlet	Inlet	Outlet	Inlet	Outlet	Inlet	Outlet	Inlet	Outlet	Inlet	Outlet	Inlet	Outlet	Inlet	Outlet	Inlet	Outlet
Don Chang	ND	0.684	0.008	0.009	0.558	0.456	0.164	0.108	ND	ND	0.007	0.024	ND	ND	0.014	0.012	0.075	0.054	ND	ND
Ban Pa Lueam	ND	0.145	0.008	0.009	0.126	0.069	0.111	0.076	0.014	ND	0.017	0.006	0.118	ND	ND	0.003	0.015	ND	0.008	0.003
Kok Samran	ND	0.046	0.010	ND	0.280	0.034	0.098	0.032	ND	0.009	0.001	0.012	ND	0.062	0.007	0.009	0.119	0.187	0.008	0.004
Ban Nong Makhuea	0.147	0.018	0.008	ND	0.253	0.076	0.274	0.043	0.005	ND	0.016	ND	0.050	ND	ND	0.004	0.159	0.015	0.006	0.003
Ban Don Po Daeng	ND	0.695	0.003	0.005	0.122	0.081	0.259	0.066	0.005	ND	0.006	ND	0.097	0.059	0.014	0.006	0.163	0.053	0.011	0.002
Ban Nong Bua Noi	0.134	-	0.003	-	0.001	-	0.020	-	0.028	-	ND	-	0.063	-	0.007	-	0.094	-	0.008	-
Ban Huai Suea Ten	ND	ND	0.002	0.009	0.053	0.079	0.187	0.153	0.011	0.011	0.007	ND	ND	0.138	ND	0.012	0.065	0.229	0.017	0.005
Kok Nam Kliang	ND	ND	0.003	0.004	1.143	0.520	0.112	0.814	0.004	ND	0.007	0.016	0.028	0.211	0.002	ND	0.081	0.077	0.006	ND
Sri Suk	1.048	0.165	0.010	0.007	0.271	0.122	0.374	0.268	ND	0.005	0.008	0.016	0.168	ND	0.011	0.012	ND	0.132	0.090	0.012
Ban Nong Tae	0.003	0.148	0.002	0.008	0.064	0.026	0.095	0.046	0.001	ND	ND	ND	0.132	0.109	0.003	0.011	0.142	0.110	0.061	0.008
Huai Sai	0.045	1.048	ND	0.008	0.057	0.052	0.073	0.039	0.001	ND	0.001	0.019	ND	0.019	0.004	0.004	0.191	0.168	0.125	0.007
Ban Sub Somboon	ND	ND	0.009	0.005	0.007	0.007	0.029	0.133	0.002	0.003	ND	0.012	0.246	0.015	ND	0.001	0.109	0.157	0.044	0.007
Na Kham	ND	-	0.018	-	0.022	-	0.015	-	0.015	-	0.017	-	0.015	-	0.015	-	0.014	-	3.269	-

‘Red’ indicates contaminant levels exceeding water quality standards; ‘ND’ means not detected via standard ICP-MS and AAS methods; ‘-’ signifies untested; Aluminum and Cobalt are not in standard criteria.

**Table 3:** Concentrations of heavy metals in water samples from various locations collected during different periods: 1st rd (transition from late hot to early rainy season; 16 July–8 August 2022), 2nd rd (mid-rainy season; 29–30 August 2022), and 3rd rd (transition from late rainy to early cool season; September–October 2022).

Metal (mg/L)	Sample Collection Point	Community Name																			
		Huai Sai		Nong Tae		Kok Nam Kliang		Nong Bua Noi		Kud Pung Kleua		Don Chang		Pa Lueam		Kok Samran		Don Po Daeng		Nong Makhuea	
		2nd rd	3rd rd	2nd rd	3rd rd	2nd rd	3rd rd	2nd rd	3rd rd	2nd rd	3rd rd	2nd rd	3rd rd	2nd rd	3rd rd	2nd rd	3rd rd	2nd rd	3rd rd	2nd rd	3rd rd
Al	Inlet	0.0054	ND	ND	ND	ND	ND	0.1023	0.078	ND	ND	ND	ND	ND	0.1502	ND	ND	0.0011	ND	ND	ND
	Precipitation Chamber	0.0476	ND	0.0128	ND	-	-	-	-	0.01	ND	ND	ND	0.0055	ND	0.0062	ND	0.0014	ND	ND	ND
	Outlet	0.0566	ND	0.0377	0.0238	ND	ND	0.0049	-	0.0159	ND	ND	ND	0.0017	ND	0.017	ND	0.0075	ND	ND	ND
Cr <0.05	Inlet	ND	ND	ND	ND	ND	ND	ND	ND	ND	ND	ND	ND	ND	ND	ND	ND	ND	ND	ND	ND
	Precipitation Chamber	ND	ND	ND	ND	-	-	-	-	ND	ND	ND	ND	ND	ND	ND	ND	ND	ND	ND	ND
	Outlet	ND	ND	ND	ND	ND	ND	ND	-	ND	ND	ND	ND	ND	ND	ND	ND	ND	ND	ND	ND
Mn <0.3	Inlet	ND	ND	ND	ND	0.5157	ND	ND	ND	ND	ND	ND	ND	ND	ND	ND	ND	ND	ND	0.0364	ND
	Precipitation Chamber	ND	0.0045	ND	ND	-	-	-	-	0.0151	0.0115	0.2191	ND	0.0141	0.0092	0.0053	0.0111	0.0131	0.2982	1.2345	0.9607
	Outlet	0.0025	ND	ND	ND	ND	ND	ND	-	0.0072	0.0192	0.0727	0.0146	0.0370	0.0841	0.0113	0.0126	0.0168	0.2973	0.3313	0.0569
Fe <0.3	Inlet	ND	ND	ND	ND	ND	ND	ND	ND	ND	ND	ND	ND	ND	0.1259	ND	0.0208	ND	0.0543	ND	ND
	Precipitation Chamber	ND	ND	ND	ND	-	-	-	-	ND	ND	ND	ND	ND	ND	ND	ND	ND	ND	ND	ND
	Outlet	ND	ND	ND	ND	ND	ND	ND	-	ND	ND	ND	ND	ND	ND	ND	ND	ND	ND	ND	ND
Co	Inlet	ND	ND	ND	ND	ND	ND	ND	ND	ND	ND	ND	ND	ND	ND	ND	ND	ND	ND	ND	ND
	Precipitation Chamber	ND	ND	ND	ND	-	-	-	-	ND	ND	ND	ND	ND	ND	ND	ND	ND	ND	ND	ND
	Outlet	ND	ND	ND	ND	ND	ND	ND	-	ND	ND	ND	ND	ND	ND	ND	ND	ND	ND	ND	ND
Cu <2.0	Inlet	ND	ND	ND	ND	ND	ND	ND	ND	ND	ND	ND	ND	ND	ND	ND	ND	ND	ND	ND	ND
	Precipitation Chamber	ND	ND	ND	ND	-	-	-	-	ND	ND	ND	ND	ND	ND	ND	ND	ND	ND	ND	ND
	Outlet	ND	ND	ND	ND	ND	ND	ND	-	ND	ND	ND	ND	ND	ND	ND	ND	ND	ND	ND	ND
As <0.01	Inlet	ND	0.0025	ND	0.0025	ND	0.0021	ND	0.0023	ND	0.0025	ND	0.002	ND	0.0026	ND	0.0027	ND	0.0022	ND	0.0027
	Precipitation Chamber	ND	0.0024	ND	0.0026	-	-	-	-	ND	0.0022	ND	0.0024	ND	0.0025	ND	0.0025	ND	0.0025	ND	0.0026
	Outlet	ND	0.0022	ND	0.0025	ND	0.002	ND	-	ND	0.002	ND	0.0024	ND	0.0025	ND	0.0021	ND	0.0021	ND	0.0025
Cd <0.003	Inlet	ND	ND	ND	ND	ND	ND	ND	ND	ND	ND	ND	ND	ND	ND	ND	ND	ND	ND	ND	ND
	Precipitation Chamber	ND	ND	ND	ND	-	-	-	-	ND	ND	ND	ND	ND	ND	ND	ND	ND	ND	ND	ND
	Outlet	ND	ND	ND	ND	ND	ND	ND	-	ND	ND	ND	ND	ND	ND	ND	ND	ND	ND	ND	ND
Pb <0.01	Inlet	ND	0.0046	ND	0.0046	ND	0.0064	ND	0.0046	ND	0.0046	ND	0.0046	ND	0.0046	ND	0.0046	ND	0.0047	ND	0.0045
	Precipitation Chamber	ND	0.0046	ND	0.0046	-	-	-	-	ND	0.0046	ND	0.0045	ND	0.0045	ND	0.0045	ND	0.0046	ND	0.0045
	Outlet	ND	0.0046	ND	0.0046	ND	0.0046	ND	-	ND	0.0046	ND	0.0045	ND	0.0051	ND	0.0045	ND	0.0045	ND	0.0045
Hg <0.001	Inlet	ND	<0.001	ND	<0.001	ND	<0.001	ND	<0.001	ND	<0.001	ND	<0.001	ND	<0.001	ND	<0.001	ND	<0.001	ND	<0.001
	Precipitation Chamber	ND	<0.001	ND	<0.001	-	-	-	-	ND	<0.001	ND	<0.001	ND	<0.001	ND	<0.001	ND	<0.001	ND	<0.001
	Outlet	ND	<0.001	ND	<0.001	ND	<0.001	ND	-	ND	<0.001	ND	<0.001	ND	<0.001	ND	<0.001	ND	<0.001	ND	<0.001

Nong Buanoi’s water system was under repair, with SCG (Siam Cement Group Co., Ltd.) providing weekly raw water replenishment. Nakhum and Kok Nam Kliang relied on groundwater. ‘Red’ indicates contamination exceeding metal standards, while ‘ND’ signifies non-detection. The dash (-) indicates tests not conducted. Aluminum and Cobalt are not included in water quality standards.

**Table 4:** Findings of health assessments, sorted by resident communities, before water improvement and LIP training.

Factors	Don Chang (n = 41)	Pa Lueam (n = 42)	Kok Samran (n = 42)	Nong Makhuea (n = 40)	Don Po Daeng (n = 41)	Nong Bua Noi (n = 44)	Huai Suea Ten (n = 39)	Nong Tae (n = 40)	Huai Sai (n = 62)	Kam Bon (n = 37)	Sub Somboon (n = 39)	Na Kham (n = 41)	Kok Nam Kliang (n = 60)
Basic information													
Age, year *	58.3 ± 7.0	62.7 ± 15.0	55.6 ± 8.7	57.1 ± 10.8	64.4 ± 9.6	61.8 ± 9.6	64.7 ± 8.0	61.7 ± 10.8	59.6 ± 11.8	63.7 ± 10.5	62.0 ± 9.3	61.3 ± 11.4	58.2 ± 12.2
Male **	8 (19.5)	7 (16.7)	8 (19.5)	15 (37.5)	13 (31.7)	12 (27.2)	12 (30.8)	11 (27.5)	14 (22.6)	13 (35.1)	13 (33.3)	10 (24.4)	19 (31.7)
Weight, kg *	64.3 ± 12.8	57.2 ± 9.4	63.3 ± 9.3	58.6 ± 9.8	59.4 ± 12.0	57.0 ± 9.2	58.6 ± 9.9	61.9 ± 13.2	62.1 ± 13.5	56.5 ± 13.5	61.4 ± 11.1	57.6 ± 8.7	60.0 ± 10.5
Height, cm *	160.9 ± 6.9	155.1 ± 6.7	154.4 ± 6.7	157.1 ± 5.9	155.4 ± 7.0	155.7 ± 8.1	151.9 ± 7.4	156.9 ± 8.0	156.6 ± 9.0	157.2 ± 7.5	153.9 ± 7.7	151.0 ± 7.5	153.9 ± 7.5
BMI, kg/m^2^ *	24.8 ± 4.5	23.8 ± 3.9	26.6 ± 4.0	23.7 ± 3.4	24.7 ± 4.9	23.5 ± 3.7	25.4 ± 3.8	25.0 ± 4.3	25.4 ± 5.3	22.8 ± 4.7	25.8 ± 3.5	25.3 ± 3.2	25.3 ± 3.9
Education **													
Not educated	0 (0.0)	2 (4.8)	0 (0.0)	0 (0.0)	0 (0.0)	2 (4.6)	1 (2.6)	0 (0.0)	0 (0.0)	2 (5.4)	1 (2.6)	0 (0.0)	1 (1.7)
Elementary education	20 (48.8)	28 (66.7)	26 (61.9)	33 (82.5)	37 (90.2)	38 (86.4)	36 (92.3)	31 (77.5)	54 (87.1)	30 (81.1)	23 (59.0)	27 (65.9)	39 (65.0)
Lower secondary education	6 (14.6)	3 (7.1)	11 (26.2)	4 (10.0)	2 (4.9)	1 (2.3)	1 (2.6)	4 (10.0)	3 (4.8)	1 (2.7)	2 (5.1)	7 (17.1)	6 (10.0)
Upper secondary education/Vocational Certificate	12 (29.3)	8 (19.1)	3 (7.1)	2 (5.0)	2 (4.9)	3 (6.8)	1 (2.6)	4 (10.0)	5 (8.1)	3 (8.1)	5 (12.8)	6 (14.6)	9 (15.0)
Associate/Higher Vocational Certificate	2 (4.9)	1 (2.4)	0 (0.0)	0 (0.0)	0 (0.0)	0 (0.0)	0 (0.0)	0 (0.0)	0 (0.0)	0 (0.0)	2 (5.1)	0 (0.0)	4 (6.7)
Bachelor’s degree	1 (2.4)	0 (0.0)	2 (4.8)	1 (2.5)	0 (0.0)	0 (0.0)	0 (0.0)	1 (2.5)	0 (0.0)	1 (2.7)	6 (15.4)	1 (2.4)	1 (1.7)
Postgraduate level	0 (0.0)	0 (0.0)	0 (0.0)	0 (0.0)	0 (0.0)	0 (0.0)	0 (0.0)	0 (0.0)	0 (0.0)	0 (0.0)	0 (0.0)	0 (0.0)	0 (0.0)
Occupation **													
Agriculture	34 (82.9)	21 (50.0)	37 (88.1)	35 (87.5)	29 (70.7)	30 (68.2)	27 (69.2)	36 (90.0)	21 (33.9)	27 (73.0)	15 (38.5)	25 (61.00	28 (46.7)
Trade or Commerce	1 (2.4)	3 (7.1)	2 (4.8)	0 (0.0)	1 (2.4)	0 (0.0)	1 (2.6)	0 (0.0)	7 (11.3)	1 (2.7)	2 (5.1)	1 (2.4)	8 (13.3)
Factory work/Company	0 (0.0)	0 (0.0)	0 (0.0)	0 (0.0)	0 (0.0)	1 (2.3)	1 (2.6)	0 (0.0)	1 (1.6)	0 (0.0)	0 (0.0)	1 (2.4)	1 (1.7)
Civil Service/State Enterprise	0 (0.0)	1 (2.4)	1 (2.4)	0 (0.0)	0 (0.0)	0 (0.0)	0 (0.0)	0 (0.0)	0 (0.0)	0 (0.0)	2 (5.1)	0 (0.0)	3 (5.0)
Currently Studying	0 (0.0)	0 (0.0)	0 (0.0)	0 (0.0)	0 (0.0)	0 (0.0)	0 (0.0)	0 (0.0)	0 (0.0)	0 (0.0)	0 (0.0)	0 (0.0)	0 (0.0)
Others	6 (14.6)	4 (9.5)	0 (0.0)	2 (5.0)	3 (7.3)	3 (6.8)	3 (7.7)	3 (7.5)	16 (25.8)	5 (13.5)	5 (12.8)	3 (7.3)	7 (11.7)
Unemployed	0 (0.0)	13 (31.0)	2 (4.8)	3 (7.5)	8 (19.5)	10 (22.7)	7 (18.0)	1 (2.5)	17 (27.4)	4 (10.8)	15 (38.5)	11 (26.8)	13 (21.7)
Monthly Income (Baht) **													
≤10,000	37 (90.2)	38 (90.5)	38 (90.5)	36 (90.0)	38 (92.7)	41 (93.2)	39 (100.0)	37 (92.5)	56 (90.3)	37 (100.0)	34 (87.2)	37 (90.2)	52 (86.7)
>10,000	4 (9.8)	4 (9.5)	4 (9.5)	4 (10.0)	3 (7.3)	3 (6.8)	0 (0.0)	3 (7.5)	6 (9.7)	0 (0.0)	5 (12.8)	4 (9.8)	8 (13.3)
Chronic diseases **													
High Blood Pressure (All)	16 (39.0)	12 (28.6)	12 (28.6)	15 (37.5)	12 (29.3)	15 (34.1)	18 (46.2)	8 (20.0)	26 (41.9)	15 (40.5)	18 (46.2)	24 (58.5)	26 (43.3)
High Blood Pressure (No diabetes)	8 (19.5)	9 (21.4)	7 (16.7)	10 (25.0)	8 (19.5)	10 (22.7)	9 (23.1)	7 (17.5)	16 (25.8)	10 (27.0)	9 (23.1)	22 (53.7)	13 (21.7)
High Blood Pressure (With diabetes)	8 (19.5)	3 (7.1)	5 (11.9)	5 (12.5)	4 (9.8)	5 (11.4)	9 (23.1)	1 (2.5)	10 (16.1)	5 (13.5)	9 (23.1)	2 (4.9)	13 (21.7)
Diabetes	10 (24.4)	4 (9.5)	5 (11.9)	7 (17.5)	5 (12.2)	8 (18.2)	10 (25.6)	1 (2.5)	11 (17.7)	8 (21.6)	10 (25.6)	4 (9.8)	13 (21.7)
Kidney Stones	1 (2.4)	0 (0.0)	0 (0.0)	2 (5.0)	0 (0.0)	2 (4.6)	1 (2.6)	0 (0.0)	1 (1.6)	2 (5.4)	0 (0.0)	0 (0.0)	0 (0.0)
Gout	2 (4.9)	0 (0.0)	1 (2.4)	1 (2.5)	1 (2.4)	0 (0.0)	3 (7.7)	1 (2.5)	1 (1.6)	3 (8.1)	1 (2.6)	3 (7.3)	1 (1.7)
Laboratory test results													
Serum creatinine level, mg/dL *	0.81 ± 0.15	0.85 ± 0.28	0.81 ± 0.26	0.89 ± 0.24	0.85 ± 0.25	0.86 ± 0.20	1.04 ± 0.71	0.82 ± 0.20	0.83 ± 0.20	0.92 ± 0.26	0.94 ± 0.34	0.82 ± 0.21	0.85 ± 0.24
Glomerular filtration rate, mL/min/1.73 m^2^ *	84.0 ± 15.9	82.0 ± 20.0	88.4 ± 17.6	83.3 ± 17.5	81.3 ± 18.2	81.3 ± 16.3	73.0 ± 21.4	85.0 ± 14.2	83.4 ± 17.4	79.9 ± 17.7	76.3 ± 20.2	82.7 ± 18.2	84.9 ± 19.1
Urine albumin/creatinine ratio, mg/g ***	5.53 (2.37–26.3)	8.89 (3.87–18.6)	6.33 (3.54–13.0)	7.99 (4.68–45.2)	9.89 (4.90–30.6)	7.16 (4.05–26.9)	11.6 (4.95–49.1)	8.77 (3.78–29.0)	10.87 (4.29–37.6)	7.83 (3.84–22.5)	7.06 (3.73–33)	7.27 (3.53–17.3)	11.59 (4.94–52.7)
CKD **	14 (34.2)	13 (31.0)	6 (14.3)	16 (40.0)	16 (39.0)	13 (29.6)	12 (30.8)	12 (30.0)	23 (37.1)	12 (32.4)	12 (30.8)	12 (29.3)	21 (35.0)
Stage 1	4 (9.8)	4 (9.5)	1 (2.4)	8 (20.1)	7 (17.1)	2 (4.6)	1 (2.6)	1 (2.5)	9 (14.5)	4 (10.8)	3 (7.7)	5 (12.2)	10 (16.7)
Stage 2	4 (9.8)	3 (7.1)	3 (7.1)	6 (15.0)	3 (7.3)	5 (11.4)	9 (22.5)	9 (22.5)	8 (12.9)	3 (8.1)	3 (7.7)	3 (7.3)	7 (11.7)
Stage 3	6 (14.6)	5 (11.9)	1 (2.4)	2 (5.0)	5 (12.2)	6 (13.6)	2 (5.0)	2 (5.0)	6 (9.7)	4 (10.8)	5 (12.8)	4 (9.8)	3 (5.0)
Stage 4	0 (0.)	1 (2.4)	1 (2.4)	0 (0.0)	1 (2.4)	0 (0.0)	0 (0.0)	0 (0.0)	0 (0.0)	1 (2.7)	1 (2.6)	0 (0.0)	1 (1.7)
Stage 5	0 (0.0)	0 (0.0)	0 (0.0)	0 (0.0)	0 (0.0)	0 (0.0)	0 (0.0)	0 (0.0)	0 (0.0)	0 (0.0)	0 (0.0)	0 (0.0)	0 (0.0)
CKDu **	5 (12.2)	7 (16.7)	2 (4.8)	6 (15.0)	10 (24.4)	3 (6.8)	2 (5.1)	9 (22.5)	9 (14.5)	4 (10.8)	1 (2.6)	4 (9.8)	7 (11.7)
CKDu, age < 70 years **	4 (9.8)	3 (7.1)	2 (4.8)	5 (12.5)	6 (14.6)	1 (2.3)	2 (5.1)	6 (15.0)	6 (9.7)	1 (2.7)	1 (2.6)	2 (4.9)	6 (10.0)
Anemia **	10 (25.0)	11 (29.0)	9 (23.1)	9 (24.3)	10 (27.0)	15 (36.6)	9 (28.1)	8 (22.2)	17 (29.8)	11 (31.4)	13 (40.6)	16 (42.1)	12 (26.1)
Hemoglobin level, g/dL *	12.6 ± 1.33	12.6 ± 1.49	12.8 ± 1.34	13.1 ± 1.35	12.60 ± 1.41	12.5 ± 2.09	12.4 ± 1.54	12.9 ± 1.27	12.9 ± 1.59	13.0 ± 1.63	12.4 ± 1.47	12.7 ± 1.40	13.0 ± 1.39
Elevated white blood cell count **	1 (2.4)	4 (10.5)	4 (10.3)	0 (0.0)	3 (8.1)	3 (7.3)	1 (3.1)	0 (0.0)	5 (8.8)	4 (11.4)	1 (3.1)	5 (13.2)	5 (10.9)
White blood cell count, cells/mL *	6709 ± 1755	7021 ± 2374	7749 ± 1767	7056 ± 1503	7497 ± 2075	7210 ± 1750	6857 ± 1934	6329 ± 1478	7588 ± 1932	7131 ± 2021	6613 ± 1926	7331 ± 2046	7621 ± 2016
Liver inflammation **	5 (12.2)	4 (10.3)	4 (10.0)	5 (13.2)	2 (5.4)	5 (11.9)	1 (3.1)	0 (0.0)	5 (8.8)	3 (8.6)	4 (12.5)	4 (10.5)	5 (10.9)
Liver enzyme levels, U/L *	21.9 ± 12.0	21.6 ± 11.4	24.1 ± 14.0	24.5 ± 11.4	20.6 ± 16.4	21.3 ± 11.2	19.2 ± 8.3	18.7 ± 6.8	20.6 ± 9.90	20.7 ± 11.8	23.1 ± 14.4	21.5 ± 14.8	22.5 ± 13.4

* Indicates values are mean ± standard deviation, ** Number (percentage), *** Median (interquartile 25th–75th percentile). CKDu stands for Chronic Kidney Disease of unknown etiology.

**Table 5:** Summary of the target communities and strategy for implementing and monitoring water quality improvement through innovation and technology.

Community Name	Health Information	Water Improvement Strategy	Innovator Numbers (Station Master)
Pa Leuam	CKD 31% CKDu 7.1% *	LIP-driven community: Empowering residents through skill development for chemical sensing and sustainable water supply management.	1
Don Chang	CKD 34.2% CKDu 9.8% *	LIP-driven community: Empowering residents through skill development for chemical sensing and sustainable water supply management.	1
Kok Samran	CKD 40% CKDu 4.8% *	LIP-driven community: Empowering residents through skill development for chemical sensing and sustainable water supply management.	2
Nong Makhuea	CKD 14.3% CKDu 12.5% *	LIP-driven community: Empowering residents through skill development for chemical sensing and sustainable water supply management.	
Don Po Daeng	CKD 39% CKDu 14.6% *	LIP-driven community: Empowering residents through skill development for chemical sensing and sustainable water supply management.	
Nong Bua Noi	CKD 29.6% CKDu 2.3% *	LIP-driven community: Empowering residents through skill development for chemical sensing and sustainable water supply management.	2
Huai Suea Ten	CKD 30.8% CKDu 5.1% *	LIP-driven community: Empowering residents through skill development for chemical sensing and sustainable water supply management.	
Nong Tae	CKD 30.6% CKDu 15.0% *	LIP-driven community: Empowering residents through skill development for chemical sensing and sustainable water supply management.	2
Huai Sai	CKD 37.1% CKDu 9.7% *	LIP-driven community: Empowering residents through skill development for chemical sensing and sustainable water supply management.	2
Kok Sung	CKDu 0% *	LIP-driven community: Empowering residents through skill development for chemical sensing and sustainable water supply management.	
Kam Bon	CKD 32.4% CKDu 2.7% *	LIP-driven community: Empowering residents through skill development for chemical sensing and sustainable water supply management.	
Na Kham	CKD 29.3% CKDu 4.9% *	LIP-driven community: Empowering residents through skill development for chemical sensing and sustainable water supply management. **Note:** using groundwater, without a community water treatment process.	2
Sub Somboon	CKD 30.8% CKDu 2.6% *	LIP-driven community: Empowering residents through skill development for chemical sensing and sustainable water supply management. **Note:** using groundwater, without a community water treatment process.	2
Kok Nam Kliang	CKD 35.0% CKDu 10% *	LIP-driven community: Empowering residents through skill development for chemical sensing and sustainable water supply management. **Note:** Using groundwater without a community water treatment process and using a village drinking water production plant for drinking water.	1
Sri Suk	ND **	LIP-driven community: Empowering residents through skill development for chemical sensing and sustainable water supply management. **Note:** Using groundwater without a community water treatment process.	2

* Data from CKDNET health exams, conducted 13–31 January 2023. ** Indicates residents opting out of health exams.

**Table 6:** Changes in health indicators and kidney function pre-and post-implementation of the LIP-driven community.

Factors	Total Participants (n = 478)		
	Before LIP	After LIP	*p*-Value
Age (Years old) *	61.17 ± 10.01	61.64 ± 9.94	<0.001
Weight (kg.), mean ± SD Median (25th–75th percentile)	60.01 ± 11.18 59 (52–67)	59.76 ± 11.47 58.85 (51.95–67)	0.17 0.002
BMI (kg/m^2^)			
Mean ± SD	24.87 ± 4.22	24.76 ± 4.27	0.13
Median (Interquartile range)	24.55 (21.91–27.18)	24.49 (21.91–27.27)	0.002
Systolic blood pressure (mmHg)	137.71 ± 18.69	126.71 ± 16.64	<0.001
Diastolic Blood pressure (mmHg)	80.71 ± 11.65	77.98 ± 10.58	<0.001
The average severity of symptoms * (Score 1–5 from no symptoms to very severe symptoms)			
Lethargy	2.74 ± 1.38	2.34 ± 1.35	<0.001
Arthralgia	3.16 ± 1.40	2.86 ± 1.44	<0.001
Muscle ache	2.85 ± 1.45	2.39 ± 1.42	<0.001
Backache	2.85 ± 1.42	2.42 ± 1.37	<0.001
Abdominal bloating	1.97 ± 1.33	1.69 ± 1.16	<0.001
Headache	2.06 ± 1.34	1.60 ± 1.06	<0.001
Loss of appetite	1.64 ± 1.16	1.49 ± 1.01	0.006
Insomnia	2.28 ± 1.45	1.97 ± 1.33	<0.001
Cramps	2.13 ± 1.39	1.79 ± 1.21	<0.001
Dysuria	1.14 ± 0.64	1.19 ± 0.67	0.19
Pale	1.12 ± 0.52	1.07 ± 0.43	0.06
Swollen face/leg	1.19 ± 0.67	1.15 ± 0.59	0.30
Jaundice	1.07 ± 0.40	1.01 ± 0.19	0.003
Palpitation	1.64 ± 1.11	1.41 ± 0.92	<0.001
Gasping for breath	1.69 ± 1.18	1.42 ± 0.93	<0.001
Diarrhea	1.42 ± 0.93	1.23 ± 0.73	<0.001
Rash	1.48 ± 1.02	1.43 ± 0.95	0.42
Other symptoms	1.14 ± 0.65	1.04 ± 0.36	0.01
Diseases found in the last 6 months **			
High blood pressure (total)	187 (39.12)	157 (32.85)	<0.001
High blood pressure (without diabetes)	119 (24.90)	97 (20.29)	0.01
High blood pressure (with diabetes)	68 (14.23)	60 (12.55)	0.001
Diabetes	80 (16.74)	84 (17.57)	0.37
Kidney stone	6 (1.26)	3 (0.63)	0.26
Gout	15 (3.14)	14 (2.93)	0.65
High cholesterol	48 (10.04)	40 (8.37)	0.19
Anemia (Thalassemia and others)	11 (2.30)	9 (1.88)	0.27
Hepatitis	3 (0.63)	1 (0.21)	0.50
Skin diseases (Discoloration)	14 (2.93)	0 (0.0)	<0.001
Gastrointestinal diseases (nausea, vomiting, diarrhea, abdominal pain)	26 (5.44)	2 (0.42)	<0.001
Neurological symptoms (numbness, weakness)	87 (18.20)	15 (3.14)	<0.001
Fatigue and lethargy	49 (10.27)	3 (0.63)	<0.001
CKD	158 (33.05)	161 (33.68)	0.79
Stage 1	46 (9.62)	31 (6.49)	
Stage 2	55 (11.51)	65 (13.60)	
Stage 3	52 (10.88)	60 (12.55)	
Stage 4	5 (1.05)	4 (0.84)	
Stage 5	0 (0.0)	1 (0.21)	
Ckdu	60 (12.55)	76 (15.90)	0.39
Ckdu, age < 70 years old	40 (8.37)	56 (11.72)	0.19
Laboratory diagnostic criteria for chronic kidney disease (n = 454)			
Mean serum creatinine level (mg/dl) *	0.85 ± 0.23	0.87 ± 0.23	<0.001
Average glomerular filtration rate (mL/min/1.73 m^2^) *	81.94 ± 17.09	80.39 ± 17.45	<0.001
Number of participants with a glomerular filtration rate less than 60 mL/min/1.73 m^2^ **	55 (12.11)	62 (13.66)	0.21
- Urine albumin/creatinine (mg/g)			
Mean ± SD	111.33 ± 462.51	52.91 ± 224.90	<0.001
Median (Interquartile range)	7.71 (3.84–26.52)	6.16 (3.52–14.2)	<0.001
- Number of participants with a urine albumin/creatinine ratio ≥ 30 mg/g ** (n = 450)	102 (22.67)	61 (13.56)	<0.001
- Number of participants with red blood cells in urine (>3 cells per high power field) ** (n = 454)	40 (8.81)	65 (14.32)	0.004
Laboratory diagnostic criteria for Hepatitis Hepatitis condition (ALT level > 36 U/L) **	44 (10.00)	28 (6.36)	0.004
Hepatitis cells (ALT, U/L) *	21.67 ± 12.20	19.99 ± 11.96	<0.001

* Indicates mean ± standard deviation; ** represents number (percentage).

**Table 7:** Regional analysis of changes in kidney function and prevalence of CKD Pre- and Post-LIP implementation.

Factors	Community Name												
	Don Chang (n = 38)	Pa Leaum (n = 35)	Kok Samran (n = 38)	Nong Makeau (n = 36)	Po Daeng (n = 33)	Nong Bua Noi (n = 29)	Hua Sua Ten (n = 35)	Nong Tae (n = 37)	Hua Sai (n = 50)	Kam Bon (n = 34)	Sub Sombun (n = 35)	Na Kham (n = 36)	Kok Nam Kliang (n = 42)
Serum Creatinine, mg/dL *													
Before LIP	0.82 ± 0.16	0.87 ± 0.29	0.81 ± 0.26	0.90 ± 0.24	0.85 ± 0.26	0.87 ± 0.23	0.90 ± 0.24	0.83 ± 0.20	0.82 ± 0.17	0.92 ± 0.27	0.90 ± 0.24	0.82 ± 0.21	0.81 ± 0.16
After LIP	0.81 ± 0.15	0.87 ± 0.30	0.84 ± 0.29#	0.93 ± 0.27#	0.88 ± 0.26	0.87 ± 0.22	0.90 ± 0.27	0.82 ± 0.19	0.85 ± 0.21	0.94 ± 0.24	0.89 ± 0.23	0.86 ± 0.19#	0.87 ± 0.19#
Glomerular filtration rate (GFR) mL/min/ 1.73 m^2^ *													
Before LIP	83.5 ± 16.1	79.9 ± 18.1	89.1 ± 17.7	82.8 ± 17.8	81.7 ± 19.0	78.3 ± 16.8	76.0 ± 18.0	83.9 ± 14.3	83.1 ± 16.5	78.6 ± 16.0	77.1 ± 18.1	82.8 ± 18.0	84.8 ± 14.3
After LIP	83.7 ± 16.0	80.8 ± 17.4	86.4 ± 16.4#	80.2 ± 19.4#	80.0 ± 18.4	79.1 ± 17.4	76.1 ± 18.7	83.5 ± 15.8	80.7 ± 18.6	76.4 ± 16.5	77.7 ± 18.0	78.6 ± 17.0#	79.8 ± 16.8#
Urinary Albumin/Creatinine Ratio, mg/g Average	129 ± 395	69 ± 229	40 ± 115	217 ± 711	65 ± 210	110 ± 361	198 ± 902	51 ± 120	150 ± 579	15 ± 20	327 ± 872	29 ± 75	69 ± 201
Before LIP	101 ± 438	32 ± 99	101 ± 387	97 ± 272	36 ± 87	41 ± 124	14 ± 19	18 ± 30	34 ± 172	12 ± 18#	161 ± 386	14 ± 29	32 ± 83
After LIP	6.40	8.12	6.33	8.07	9.89	7.08	7.35	8.13	8.35	6.74	5.25	7.27	11.36
Median	(2.88–27.64)	(2.96–18.61)	(3.55–11.25)	(4.68–45.18)	(4.67–27.56)	(4.25–26.9)	(4.91–17.43)	(3.96–29.03)	(4.40–31.16)	(3.84–19.07)	(3.49–33)	(3.00–20.17)	(4.96–53.23)
Before LIP	3.48#	7.10	5.14	6.23#	6.57#	8.65	5.91#	6.15#	4.75#	7.59	6.14#	6.12#	8.34#
After LIP	(2.38–8.28)	(3.94–15.85)	(3.33–12.22)	(3.34–25.62)	(3.46–19.0)	(3.9–15.5)	(4.13–18.35)	(3.34–18.71)	(3.25–9.48)	(4.41–12.99)	(3.33–36.99)	(3.81–12.31)	(4.16–15.30)
CKD before LIP **	13 (34.2)	14 (40.00)	6 (15.79)	12 (33.33)	14 (42.42)	9 (31.03)	10 (28.57)	12 (32.43)	19 (38.00)	10 (29.41)	11 (31.43)	12 (33.33)	16 (38.10)
Stage 1	3	4	1	5	6	0	1	1	7	2	3	5	8
Stage 2	4	3	3	5	2	3	3	9	7	4	3	3	6
Stage 3	6	6	1	2	5	6	5	2	5	3	5	4	2
Stage 4	0	1	1	0	1	0	1	0	0	1	0	0	0
Stage 5	0	0	0	0	0	0	0	0	0	0	0	0	0
LIP after LIP **	11 (28.95)	15 (42.86)	8 (21.05)	14 (38.89)	13 (39.39)	12 (41.38)	12 (34.29)	7 (18.92)	20 (40.00)	11 (32.35)	15 (42.86)	10 (27.78)	13 (30.95)
Stage 1	4	3	2	3	2	2	3	2	3	1	2	2	2
Stage 2	4	7	5	6	6	5	3	4	9	3	6	3	4
Stage 3	3	4	0	5	4	5	5	1	8	7	6	5	7
Stage 4	0	1	1	0	1	0	1	0	0	0	0	0	0
Stage 5	0	0	0	0	0	0	0	0	0	0	1	0	0
CKDu age < 70 years old **													
Before LIP	4 (10.53)	3 (8.57)	2 (5.26)	4 (11.11)	6 (18.18)	0 (0.0)	2 (5.71)	6 (16.22)	4 (8.00)	1 (2.94)	1 (2.86)	2 (5.56)	5 (11.90)
After LIP	4 (10.53)	6 (17.14)	4 (10.53)	7 (19.44)	8 (24.24)	2 (6.90)	4 (11.43)	1 (2.70) #	5 (10.00)	5 (14.71) #	1 (2.86)	2 (5.56)	7 (16.67)

Note: # Indicates *p* < 0.05; * Indicates mean ± SD; ** represents number (percentage).

## Data Availability

The data supporting the reported results were generated from health and water contaminant surveys, including blood and urine tests. Due to privacy and ethical restrictions, these data are not publicly available.
